# Microstructure and High-Temperature Wear Performance of FeCr Matrix Self-Lubricating Composites from Room Temperature to 800 °C

**DOI:** 10.3390/ma13010051

**Published:** 2019-12-20

**Authors:** Gongjun Cui, Yanping Liu, Guijun Gao, Huiqiang Liu, Ziming Kou

**Affiliations:** 1 College of Mechanical and Vehicle Engineering, Taiyuan University of Technology, Taiyuan 030024, China; yanpingliutyut@sina.com (Y.L.); guijungao333@163.com (G.G.); huiqiang1990@163.com (H.L.); kzmingtyut@163.com (Z.K.); 2 National-Local Joint Laboratory of Mining Fluid Control Engineering, Taiyuan 030024, China

**Keywords:** FeCr matrix composites, high temperature, friction, wear, CuO

## Abstract

FeCr matrix high-temperature self-lubricating composites reinforced by Mo, Ag, and CuO were fabricated by the powder metallurgy technique. The tribological behaviors of composites were studied at temperatures up to 800 °C. The CuO content was optimized according to the tribological results. Mo showed an obvious lubricating effect when it converted into MoO_3_. The bimetallic oxide system formed high-temperature solid lubricants with low shear strength. CuO reacted with MoO_3_ and formed CuMoO_4_ and Cu_3_Mo_2_O_9_. The composites showed an increase in the friction coefficient with the increase of CuO. However, the wear rates decreased with the increase of CuO. The critical threshold at which there was a transition of friction coefficients and wear rates from room temperature (RT) to 800 °C was 10 wt.% CuO. The Fe(Cr)-14% Mo-10.5% Ag-10% CuO composite showed the most reasonable high-temperature tribological behaviors. This was ascribed to the synergistic effects of silver, Mo, in situ formed solid lubricants (metal oxides and salt compounds), and the stable oxide film on the worn surfaces. At elevated temperatures, the dominant wear mechanism was oxidation wear.

## 1. Introduction

High-temperature wear usually leads to the loss of mechanical strength and to a decrease in the service life of mechanical parts [1,2,3]. Nowadays, high-temperature mechanical parts are made of nickel and cobalt matrix materials due to their excellent oxidation resistance and mechanical strength. In some cases, ceramic matrix materials are also used under some rigorous working conditions. Although these materials have a considerable wear resistance at elevated temperatures, the cost is high. It is therefore necessary to develop composites with a low cost, a low friction coefficient, and a high wear resistance at elevated temperatures. Fe matrix material is one of the superalloys, which are widely used in many industrial fields as wear resistant mechanical parts [4,5]. High-temperature Fe matrix sleeves and bears always run at 600 °C. However, wear resistance depends on the mechanical properties of materials at elevated temperatures [6,7], resulting in severe wear. Therefore, Fe matrix materials with a low cost and wear rate are promising materials for tribological application, providing strong motivation for engineers to improve the wear performance of Fe matrix materials to replace nickel and ceramic matrix materials at high temperatures.

The hardfacing of Fe alloys is a popular means to change the surface composition, so as to improve the wear properties, by using C and B elements [8,9]. The hard Fe(B,C) phase can carry part of the normal load during the wear process in order to decrease the wear rate. However, brittleness is the inherent nature of the Fe(B,C) phase, which easily fractures and peels off from the worn surface, destroying the wear resistance of alloys [10]. The surface modification methods do not change the bulk properties, which restricts their application as bulk materials. Kim et al. [11] reported the effect of Mn on the wear behaviors of Fe-20Cr matrix hardfacing alloys from 25 to 450 °C. The results indicated that γ→ε martensitic transformation was beneficial to the wear resistance of alloys when Mn content exceeded 10 wt.%. The excellent wear resistance of Fe matrix alloys could be achieved by adding hard ceramic particles, such as SiC, TiC, and Al_2_O_3_ [12,13,14]. Zhang et al. [15] prepared Fe-28Al-5Cr matrix composites reinforced by nano-TiC ceramic particles. High TiC content increased the wear resistance of the Fe–Al–Cr alloy at 800 °C. However, TiC ceramic particles obviously destroyed the friction coefficient of composites—the friction coefficient was about 0.7. Solid lubricants work as an effective strengthening phase to reinforce the tribological properties of metal matrix composites at elevated temperatures [16,17,18]. During sliding, solid lubricants form a stable lubricating film on the contact surfaces in order to provide a lubricating effect for composites. Song et al. [19] investigated the wear and friction behaviors of a CuO/3Y-TZP composite. CuO reacted with Al_2_O_3_ and formed CuAl_2_O_4_ and CuAlO_2_. The CuAlO_2_ formed a soft surface layer which could reduce friction coefficients. Ba_0.25_Sr_0.75_SO_4_ showed a good lubricating effect in Fe_3_Al alloys [20,21]. Ba_0.25_Sr_0.75_SO_4_ softened and formed a lubricating film on the contact surfaces at elevated temperatures. However, the hardness and compactness of Fe_3_Al matrix composites degraded due to the addition of Ba_0.25_Sr_0.75_SO_4_ [20]. In our previous work [22], BaF_2_ was used to improve the wear resistance of FeCr matrix composites as high-temperature solid lubricants. The glaze film containing BaF_2_ and different compounds played an important part in the tribological properties. FeCr with 10% BaF_2_ showed an excellent wear resistance from RT to 800 °C. Nevertheless, the wear rates were in the order of 3.5 × 10^−4^ mm^3^/N.m. We thought that FeCr matrix self-lubricating composites could be further improved by adopting appropriate means.

In this research project, FeCr alloying powder was selected as a matrix. Ag, Mo, and CuO were used to reinforce the tribological behaviors of FeCr matrix composites, and the CuO content was optimized. The powder metallurgy technique (P/M) was employed for preparing composites. Wear tests were carried out using a ball-on-disk high-temperature tribotester from room temperature (24 °C) to 800 °C. The corresponding friction mechanisms were explored.

## 2. Experimental Procedure

### 2.1. Specimens

Fe (purity 99.5, 75 μm, Sinopharm Chemical Reagent Co., Ltd., Shanghai, China) and Cr (purity 99.3, 60 μm, Sinopharm Chemical Reagent Co., Ltd., Shanghai, China) powders were used as the raw materials to prepare Fe-21 wt.% Cr alloying powder. Firstly, Fe and Cr powders were mixed in a planetary ball mill and then sintered at 1100 °C in a furnace. The alloying powder was milled by a high-energy mill for 12 h, and the rotational speed was 300 rpm [22]. The mean size of the Fe-21 wt.% Cr alloying powder was about 40 μm. The sizes of the Ag, Mo, and CuO powders (Sinopharm Chemical Reagent Co., Ltd., Shanghai, China) used were about 65, 25, and 75 μm, respectively. The different powders were weighted according to Table 1. The composites were denoted as FC, FU, FM, FAC10, FAC6, and FAC14. The raw materials were mechanically mixed together by a high-energy mill for 8 h, and the rotational speed was 150 rpm. The ratio of powder to ball was 8:1. Mixtures were put into a graphite die (inner diameter: 30 mm). Composites were sintered under a pressure of 35 MPa for 30 min at 1150 °C in a vacuum hot-pressing furnace.

### 2.2. Tribological Tests

The wear and friction performances were evaluated on a tribometer (Zhongke Kaihua Co., Ltd., Lanzhou, China) with a ball-on-disk configuration from 24 to 800 °C in air. The counterpart was a Si_3_N_4_ ceramic ball with a diameter of 6 mm, and its hardness was about 15 GPa (Hv). The ceramic ball was fixed. FeCr matrix composites were cut into disks with a size of ⌀ 30 mm × 4 mm. Before the tests, the testing surfaces were polished to an average roughness of 0.34 μm (R_a_) and the disks were rotated. The testing speed and normal load were 0.19 m/s and 10 N, respectively. The duration of the test was 20 min, and the turning radius was 5 mm. The testing temperatures were room temperature (24 °C), 200, 400, 600, and 800 °C. Each experimental point was repeated three times in order to ensure the accuracy of the data. The friction coefficients of the composites were recorded online by a computer.

The microstructures and worn surfaces of the composites were analyzed using scanning electron microscopy (SEM, IT-300, JEOL, Tokyo, Japan) and energy dispersive spectroscopy (EDS, Inca X-MAX-100, Oxford, England). The phases of the composites were characterized by X-ray diffraction (XRD, DIFFRACTOMETER-6000, 40 kV, 30 mA, Cu Ka radiation, Tokyo, Japan). Archimedes’ method was explored to determine the density of the testing specimens. The hardness was measured using a Vickers indentation (MH-5 micro-hardness tester, Suzhou, China). The test load was 200 g, and the dwell time was 10 s. The hardness reported here was the average value of ten measurements. The wear depth profiles of the wear tracks were examined by a contact surface profiler to calculate the wear volume of composites. The specific wear rate was calculated as the wear volume (mm^3^) divided by the sliding distance (m) and the applied load (N), and its unit was mm^3^/N.m.

## 3. Results and Discussion

### 3.1. Microstructure and Physical Properties of Composites

**Figure 1:**
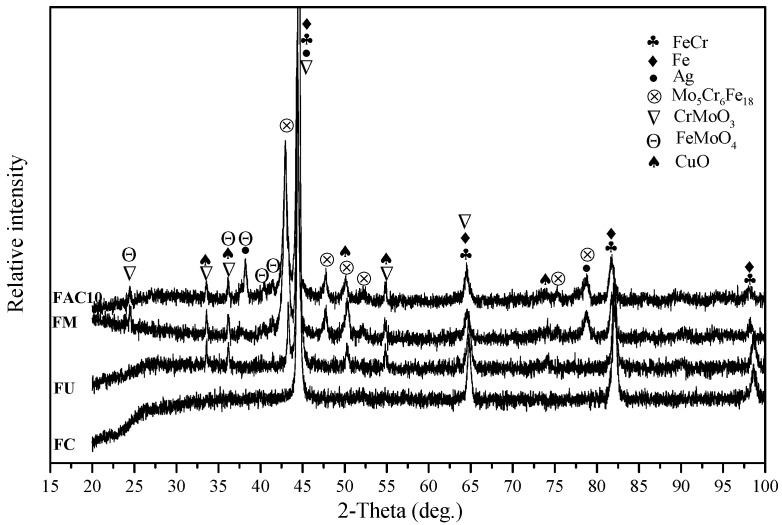
XRD patterns of obtained composites.

The XRD patterns of the obtained FeCr matrix composites are given in Figure 1. The Fe element reacts with Cr and forms a FeCr phase due to the high-temperature solid solution reaction at an elevated temperature [23]. With the addition of Mo, some new diffraction peaks were detected from the specimens, such as the CrMoO_3_, FeMoO_4_, and Mo_5_Cr_6_Fe_18_ phases. This is ascribed to the complex high-temperature reaction between Mo and other elements during solidification. These phases are expected to improve the mechanical and tribological properties of composites. Therefore, the following chemical reactions are reasonable:

Fe + Cr → FeCr(1)

7Mo + 19Fe + 7Cr + 7/2O_2_ → CrMoO_3_ + FeMoO_4_ + Mo_5_Cr_6_Fe_18_ (2)

According to the XRD pattern, the peaks of CuO are obvious, indicating that the CuO showed a high stability in the composites. Silver was present in its simple substance phase in the matrix. For FAC6, FAC10, and FAC14, the main phases consisted of Fe, FeCr, CrMoO_3_, FeMoO_4_, Mo_5_Cr_6_Fe_18_, CuO, and Ag.

**Figure 2:**
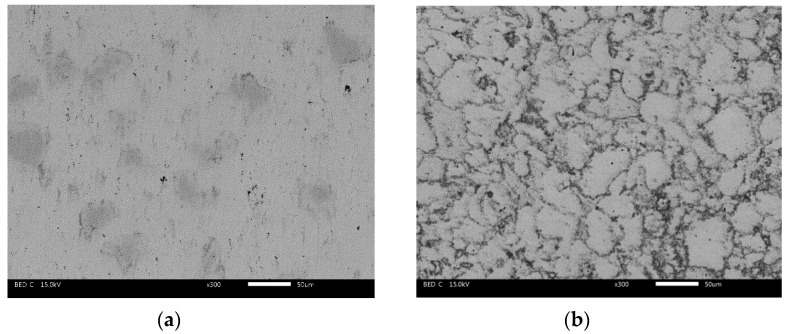

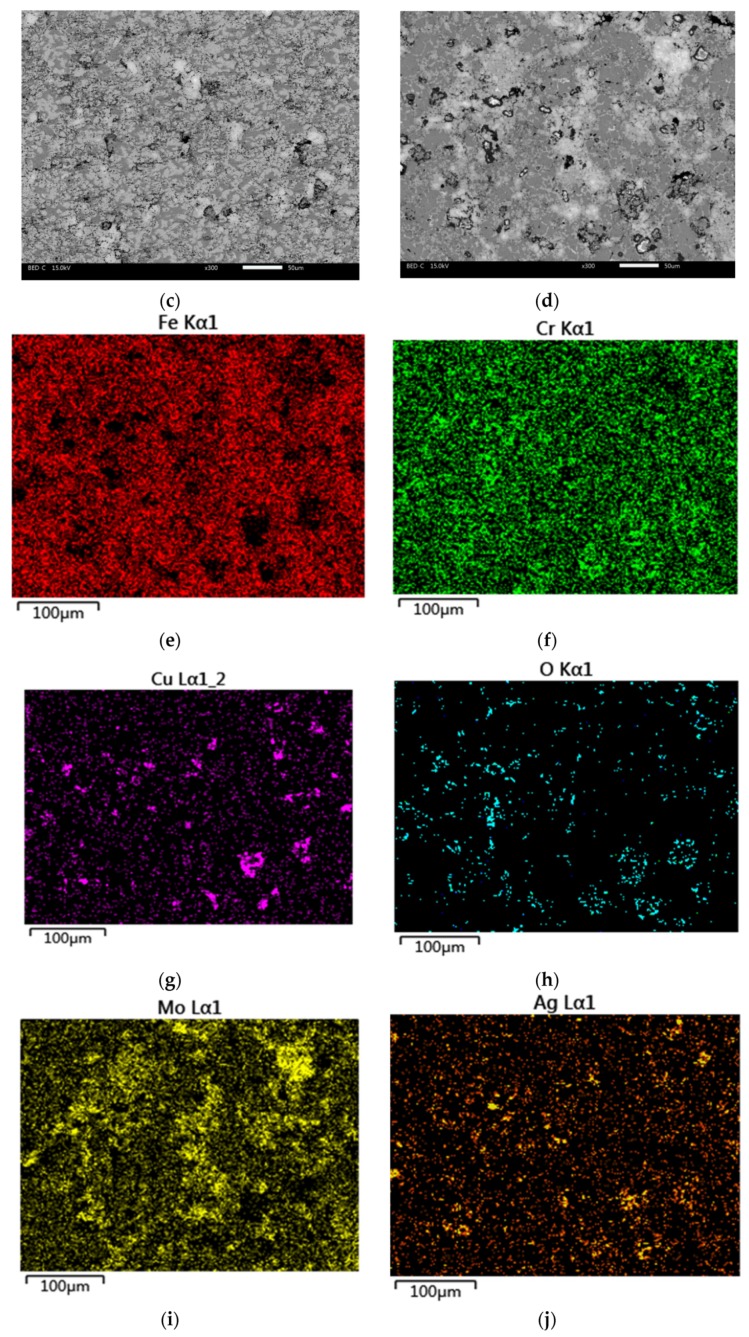
Microstructures of obtained composites: (**a**) FC, (**b**) FU, (**c**) FM and (**d**) FAC10; EDS element distribution maps of FAC10: (**e**) Fe, (**f**) Cr, (**g**) Mo, (**h**) O, (**i**) Ag and (**j**) Cu.

Figure 2 illustrates the typical microstructures and element distribution maps of FC, FU, FM, and FAC10. The figure shows that the composites had a dense microstructure, which is of benefit to the mechanical properties of the composites. The composites showed different microstructures because of differences in their composition. CuO was mainly distributed at the interface of FeCr metal particles (see Figure 2b), and CuO formed a network structure in the matrix. Mo can be seen as a bridge of metal particles when it is distributed along the interface. During solidification, Mo can react with FeCr metal particles and form intermetallic compounds induced by atom diffusion at the interface, which leads to the formation of a sintering neck of metal particles in the matrix. In addition, with the formation of a sintering neck, part of the CuO phrase was squeezed out from the interface of metal particles and showed an aggregation phenomenon (see Figure 2c). Due to this, the continuity of the matrix was improved. With increasing temperature, silver filled the gaps in the matrix because of its excellent toughness. The CuO was further squeezed out of the interfaces. Meanwhile, the chemical reaction accelerated, which reinforced the sintering neck of the metal particles and improved the continuity of the matrix in comparison with those of FU and FM. Additionally, the aggregation phenomenon of CuO became more and more obvious (see Figure 2d). Taking EDS element distribution maps and XRD patterns into account, the gray area is the mixed phases of Fe and FeCr. The Mo-rich phase is the light-gray area that contains complex compounds (see Figure 1 and Figure 2g). Silver and CuO were dispersed in the matrix (see Figure 2i,j).

Table 2 gives the hardness, density, and porosity of specimens. Although the CuO phase destroys the continuity of the matrix, CuO can restrict the slip of dislocation and the propagation of a crack as the second phase when the deformation occurs. In this case, CuO possessed an obvious particle strengthening effect on the matrix. Therefore, the hardness of composites increases with increasing CuO content. Mo formed intermetallic compounds and salt compounds in the matrix. This indicates that Mo has a solid solution strengthening effect on composites. Meanwhile, the addition of Mo improves the formation of the sintering neck of FeCr metal particles. Based on these two factors, the hardness and compactness of composites further increase. The silver, with a low hardness, was dispersed in the matrix and influenced the continuity of the matrix to some degree. Silver leads to a decrease in the hardness of composites. Nevertheless, the hardness of the specimens with silver were higher than that of other specimens. The hardness of the composites was about 1.3 times higher than that of composites without solid lubricants. Concerning density, it is easy to explain why the sintered density of the composites decreased with the increase of low density CuO—due to the wettability of the CuO phase and metal matrix, small voids could accumulate at the interfaces. As a result, the porosity increased slightly with increasing CuO content.

### 3.2. High-Temperature Tribological Properties

**Figure 3:**
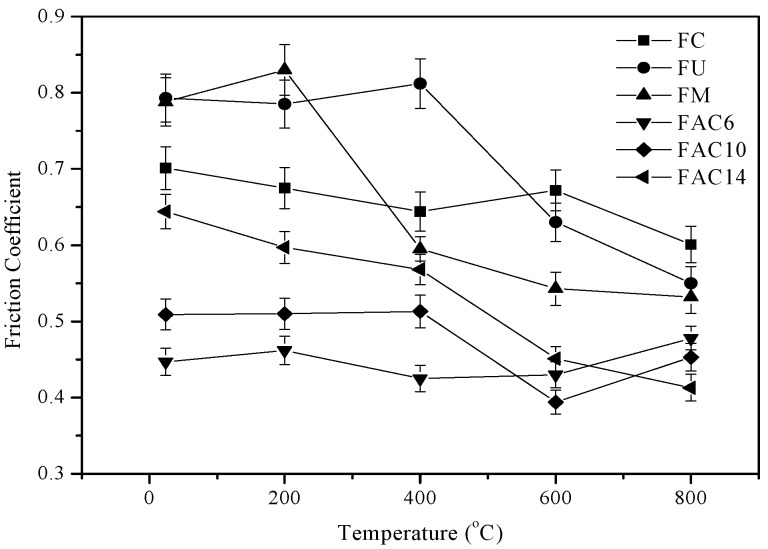
Variations of friction coefficients of the obtained composites with temperature at 10 N and 0.19 m/s.

The friction coefficients of specimens at different temperatures sliding against a Si_3_N_4_ ball are shown in Figure 3. The friction coefficients of composites generally decreased as temperature increased. At 600 and 800 °C, FU had a lower friction coefficient than that of FC. The friction coefficients of FM were lower than those of specimens FC and FU from 400 to 800 °C. Due to the addition of silver, the friction coefficients of composites decreased at low temperatures. FA6, FA10, and FA14 with CuO and silver kept low friction coefficients from RT to 800 °C, and the friction coefficients increased with increasing CuO content below 600 °C. At 800 °C, the friction coefficients show the opposite trend.

**Figure 4:**
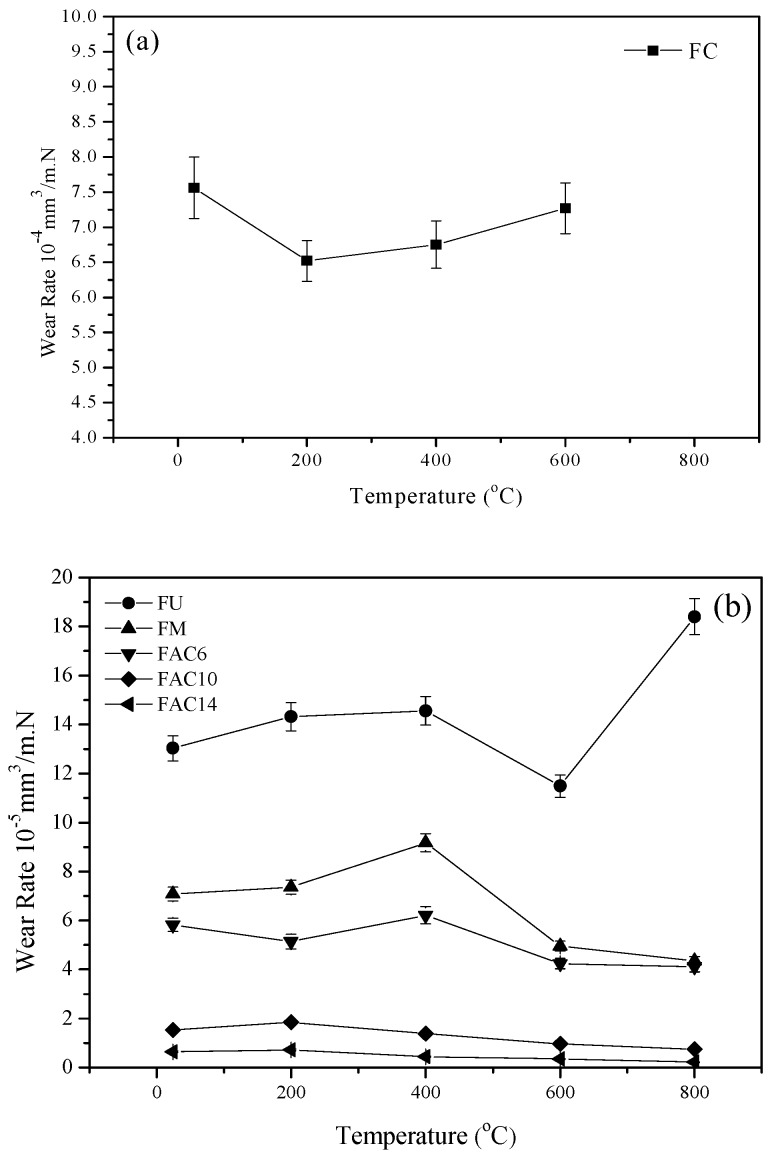
Variations of the wear rates of the obtained composites with temperature at 10 N and 0.19 m/s: (**a**) FC and (**b**) FU, FM, FAC6, FAC10, and FAC14.

Figure 4 illustrates the vibrations of the wear rates of specimens at different temperatures sliding against a Si_3_N_4_ ball. The wear rates of different composites show different trends from 24 to 800 °C. FC had the highest wear rates. Especially, at 800 °C, the wear rate was about 3.4 × 10^−2^ mm^3^/N.m. This means that 800 °C exceeds the working temperature of the FeCr material. It is clear that the wear rates of composites decrease due to the addition of Mo, CuO, and Ag. The wear rates of FM, FAC6, and FAC10 were in the order of 10^−5^ mm^3^/N.m and that of FAC14 was in the order of 10^−6^ mm^3^/N.m. FAC14 had the lowest wear rates from 24 to 800 °C. The working temperature of modified FeCr matrix composites can reach up to 800 °C. The wear rates of composites containing CuO were about 15−100 times lower than that of the specimen with CaF_2_ from room temperature to 800 °C [22]. The wear mechanism is discussed below.

**Figure 5:**
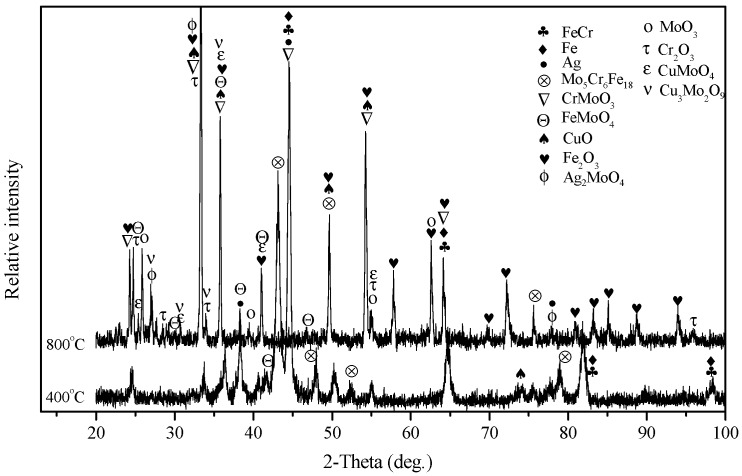
XRD patterns of the worn surfaces of FAC10 at 400 and 800 °C.

**Figure 6:**
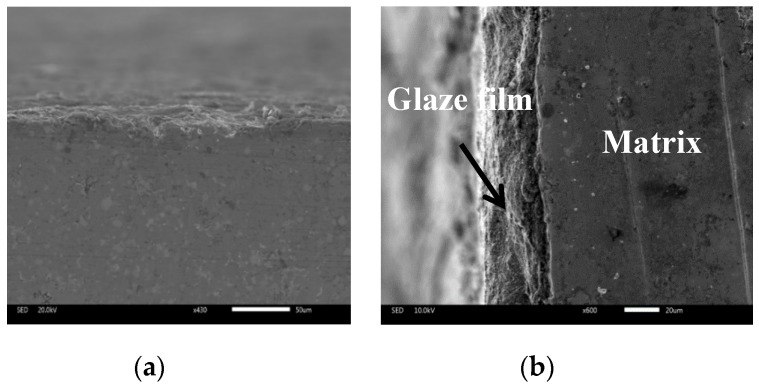
SEM images of cross sections for FAC10 at (**a**) 400 °C and (**b**) 800 °C.

Temperature is an important factor for the high-temperature friction and wear behaviors of materials [24,25,26]. At elevated temperatures, metal elements and wear debris are easily oxidized in order to produce a large amount of oxides on the wear tracks (see Figure 5). These oxides and compounds were ground by the tribo-couples and formed an obvious oxide film on the wear tracks. During sliding, the oxide film reduces the real contact area of tribo-couples and can provide a lubricating effect for the composites at elevated temperatures. When the testing temperature was low, the oxide content was low and there was no obvious oxide film on the wear tracks (see Figure 6). Thus, the friction coefficients and wear rates of composites decrease when the temperature increases. CuO does not have a lubricating effect at low temperatures. At low temperatures, CuO increases the sliding resistance in order to increase the friction coefficients of the composites as a third body. This trend becomes more and more obvious with increasing CuO content. However, at high temperatures, CuO shows a lubricating effect on composites as a high-temperature solid lubricant. Erdemir et al. [27] established an ionic potential (*Φ* = *Z*/*r*) lubricating effect model of metal oxides at elevated temperatures. They found that metal oxides with a high ionic potential have good shear performance, i.e., the oxides have a good lubricating effect. Additionally, they also investigated dual systems of metal oxides as solid lubricants and found that the greater the difference of the ionic potential of two metal oxides, the better the lubricating effect. In this study, the ionic potential of MoO_3_ was 8.95. Therefore, MoO_3_ can show a lubricating effect to some extent. The differences of MoO_3_–CuO (2.73), MoO_3_–Cr_2_O_3_ (4.84), MoO_3_–Fe_2_O_3_ (5.45), and MoO_3_–Ag_2_O (0.79) were 6.22, 4.11, 3.5, and 8.16, respectively. MoO_3_–CuO, MoO_3_–Cr_2_O_3_, MoO_3_–Fe_2_O_3_, and MoO_3_–Ag_2_O systems can show excellent lubricating effects due to the formation of CuMoO_4_, FeMoO_4_, Cu_3_Mo_2_O_9_, and Ag_2_MoO_4_ during sliding [27,28]. The Mo element is not a solid lubricant as an alloying element. However, Mo shows a lubricating effect when Mo produces CrMoO_3_, FeMoO_4_, and MoO_3_ [22,28]. Therefore, the specimens with Mo kept low friction coefficients and wear rates at elevated temperatures. Silver is an effective low-temperature solid lubricant because of its excellent ductility [29]. At high temperatures, Ag provides lubrication when it changes into Ag_2_MoO_4_ [27]. Due to the effect of Ag and Ag_2_MoO_4_, the wear and friction of the composites with silver further decreased from RT to 800 °C. The mechanical properties of materials also determine the wear rates of materials. The hardness of materials is the most important factor [30]. Materials with a high hardness have low wear rates. With the addition of CuO and Mo, the hardness of composites increases in order for the wear rates of composites to decrease. Although the specimens containing silver and CuO had a low hardness in comparison with that of FM, the in-situ formed solid lubricants, oxide film, and adding solid lubricants compensated for the influence of hardness. Therefore, FAC6, FAC10, and FAC14 kept low friction coefficients and wear rates from room temperature to 800 °C. FAC14 had the lowest wear rates but had a high friction coefficient. The obtained results indicate that 10 wt.% CuO is a critical threshold at which there is a transition of friction coefficients and wear rates. In general, FAC10 had the most reasonable tribological properties from room temperature to 800 °C. Therefore, the operating temperature of reinforced materials could reach 800 °C.

### 3.3. Analysis of Worn Surfaces

**Figure 7:**
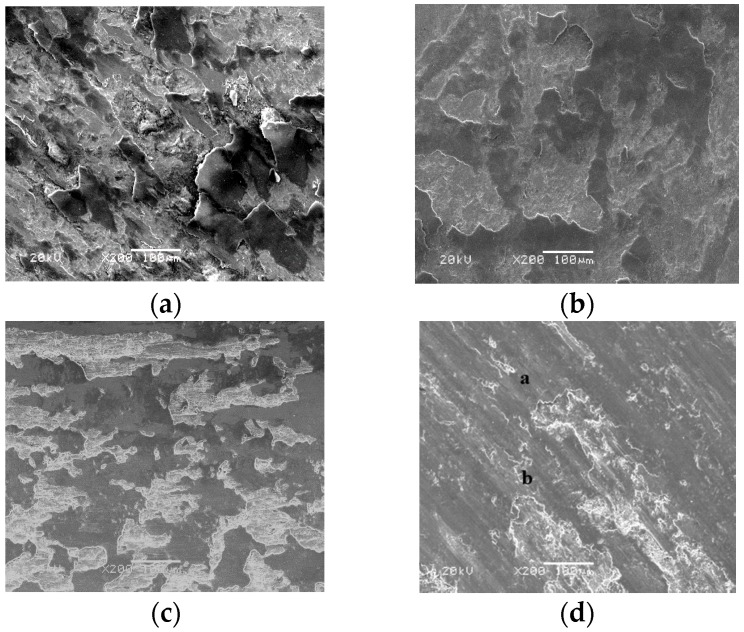
SEM images of the wear morphologies of different composites at room temperature: (**a**) FC, (**b**) FU, (**c**) FM, and (**d**) FAC10.

**Figure 8:**
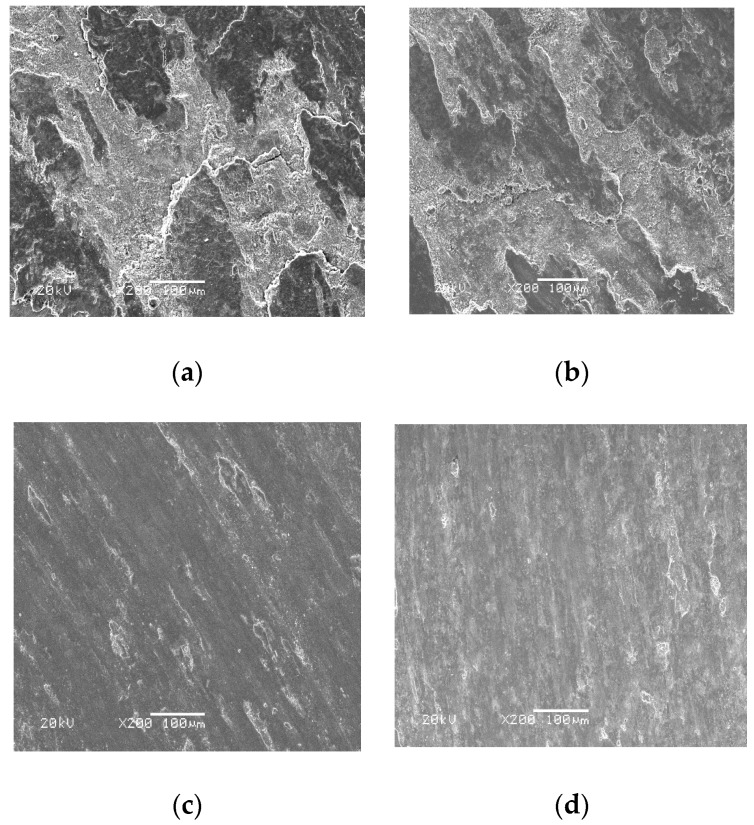
SEM images of the wear morphologies of different composites at 400 °C: (**a**) FC, (**b**) FU, (**c**) FM, and (**d**) FAC10.

**Figure 9:**
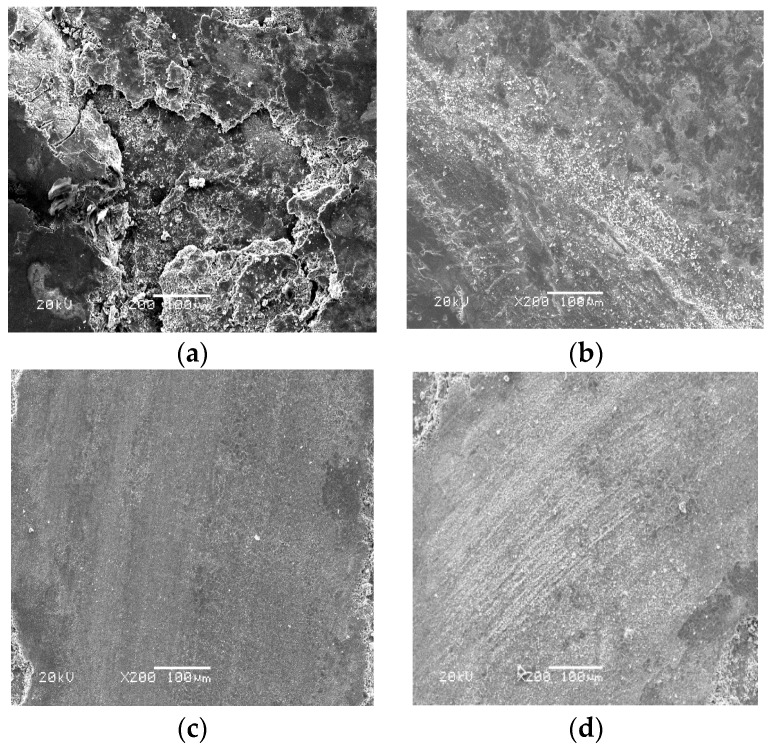
SEM images of the wear morphologies of different composites at 800 °C: (**a**) FC, (**b**) FU, (**c**) FM, and (**d**) FAC10.

Figure 7 gives the worn morphologies of FC, FU, FM, and FAC10 at RT sliding against a Si_3_N_4_ ball. The worn surfaces of FC, FU, and FM were characterized by flaking pits and a smooth area at room temperature (see Figure 7a–c). During sliding, the hardness of the Fe phase in the matrix decreases due to the friction heat [23]. Firstly, the Fe phase deforms plastically. The alloying phases (Fe(Cr,Mo)) with high hardness could support external loads and restrict the extension of plastic deformation, resulting in the size of the flaking pits decreasing with the addition of CuO and Mo. This indicates that the wear mechanisms of FC, FU, and FM were plastic deformation and fatigue wear at RT. FAC10 showed a distinct morphology compared with those of other specimens (see Figure 7d). The wear track presented slight grooves, deformation, and flaking pits. Silver with high toughness can be smeared on the wear tracks (see Table 3) to provide a lubricating effect in order to reduce the friction heat [27]. The dominant wear mechanism of FAC10 was plowing with slight fatigue wear at room temperature. At 400 °C, FC and FU showed characteristics of severe plastic deformation and flaking pits (see Figure 8a,b). This also corresponds to the high wear rates of composites at 400 °C. The worn surfaces of FM and FAC10 were smoother than those of FC and FU (see Figure 8c,d). The in situ formed lubricants started to provide a lubricating effect for the composites at 400 °C. The wear mechanisms of FM and FAC10 were characterized by plowing and slight plastic deformation. FC suffered from severe wear at 800 °C, the worn surface was completely destroyed (see Figure 9a), and a crack and delamination were noted on the worn surface. This means that FC had a high wear rate at 800 °C. In other cases, the solid lubricants, oxides, and compounds form an obvious lubricating film on the worn surfaces [24,31] (see Figure 9b–d). Additionally, the substrate with high hardness can support the formation of lubricating film on the contact surfaces. This film can protect the surfaces and restrict the process of oxidation–oxide-removal–oxidation. Meanwhile, the lubricating film also reduces the contact area of tribo-couples, so as to improve the tribological performance of composites at elevated temperatures [31]. The main wear mechanism of the obtained composites was oxidation wear with slight plowing at 800 °C.

**Figure 10:**
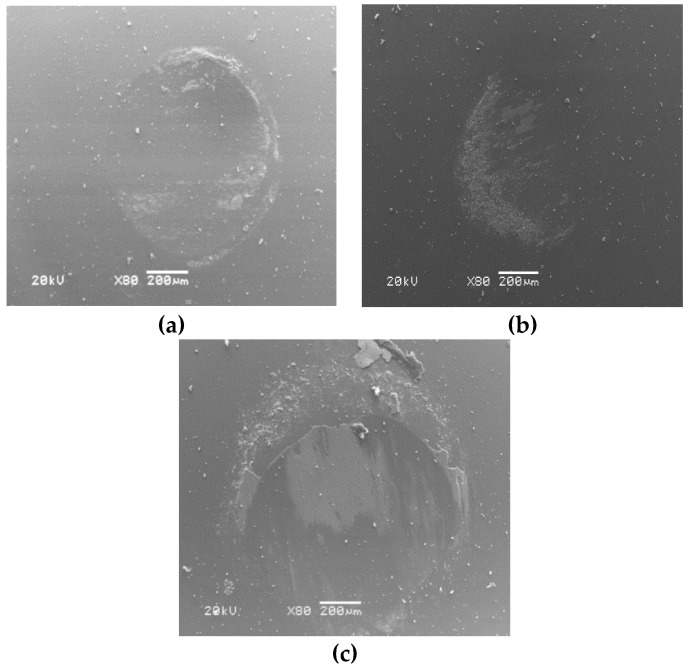
Wear morphologies of Si3N4 balls sliding against FAC10 at different temperatures: (**a**) 24 °C, (**b**) 400 °C, and (**c**) 800 °C.

The worn surfaces of the Si_3_N_4_ balls sliding against FAC10 at different temperatures are shown in Figure 10. It is clear from the figure that a transferred layer was found on the wear scars of balls from 24 to 800 °C, and the transferred layer increased with increasing testing temperatures. The transferred layer can protect the tribo-couples during friction. The presence of a transferred layer can change the contact model between the composites and the ceramic balls to reduce wear and friction at high temperatures.

## 4. Conclusions

(1) The addition of Mo, Ag, and CuO greatly changed the microstructure, phases, and hardness. The continuity of the matrix was improved. The distribution of CuO transferred from a network to an agglomeration state in the matrix. The Mo element showed a solid solution strengthening effect and a lubricating effect when it changed into CrMoO_3_, FeMoO_4_, CuMoO_4_, Cu_3_Mo_2_O_9_, Ag_2_MoO_4_, and MoO_3_. CuO had a particle strengthening effect on the matrix. The hardness of the composites was about 1.3 times higher than that of composites without solid lubricants;

(2) Composition significantly influenced the tribological properties of composites. The friction coefficients increased with increasing CuO content. At 800 °C, the composite with a high CuO content had the lowest friction coefficient. The wear rates decreased with the increase of CuO. This was ascribed to the in situ formed lubricants (CrMoO_3_, FeMoO_4_, CuMoO_4_, Cu_3_Mo_2_O_9_, Ag_2_MoO_4_, and MoO_3_), silver, CuO, and the stable oxide film. Additionally, the hardness of composites also played an important part in the wear resistance. The critical threshold at which there was a transition of tribological behaviors was 10 wt.% CuO. Fe(Cr)-14% Mo-10.5% Ag-10% CuO showed the most reasonable tribological properties from 24 to 800 °C. The wear rates were about 35 times lower than those of FeCr material. The prepared FeCr matrix self-lubricating composites could replace nickel and ceramic matrix composites;

(3) MoO_3_–CuO, MoO_3_–Cr_2_O_3_, MoO_3_–Fe_2_O_3_, and MoO_3_–Ag_2_O systems can show excellent lubricating effects due to the formation of CuMoO_4_, FeMoO_4_, Cu_3_Mo_2_O_9_, and Ag_2_MoO_4_ during sliding. Bimetallic oxides with a high ionic potential difference formed high-temperature solid lubricants;

(4) The wear mechanisms of the composites were plastic deformation and fatigue wear at low temperatures. At elevated temperatures, the dominant wear mechanism was oxidation wear.

## Figures and Tables

**Table 1:** Composition of composites (mass%).

Specimens	Fe-21Cr	Mo	Ag	CuO
FC	100	0	0	0
FU	90	0	0	10
FM	76	14	0	10
FAC10	65.5	14	10.5	10
FAC6	69.5	14	10.5	6
FAC14	61.5	14	10.5	14

**Table 2:** Vickers hardness, density, and porosity of obtained composites.

Specimens	Vickers Hardness	Density (g/cm^3^)	Porosity (%)
FC	176	7.69	0.43
FU	192	7.44	1.91
FM	445	7.68	1.76
FAC10	403	7.94	1.37
FAC6	386	8.04	0.86
FAC14	428	7.83	1.94

**Table 3:** Composition of Areas a and b in Figure 7d.

Area	Element (wt.%)							
	N	O	Si	Cr	Fe	Cu	Mo	Ag
a	0.31	24.35	0.78	9.17	21.11	13.35	9.42	21.51
b	0.28	25.31	0.64	9.84	22.63	10.51	10.42	20.37
